# A Novel Splicing Mutation Leading to Wiskott-Aldrich Syndrome from a Family

**DOI:** 10.1155/2024/2277956

**Published:** 2024-02-19

**Authors:** Lingyu Wang, Jie Zhang, Linna Lu, Juan Ren, Yaofang Zhang, Lidong Zhao, Wukang Shen, Xucheng Hu, Shuai Fang, Xiaomei Lu, Gang Wang, Linhua Yang

**Affiliations:** ^1^Department of Biochemistry and Molecular Biology, Shanxi Medical University, Shanxi, China 030607; ^2^First Hospital of Shanxi Medical University, Shanxi, China 03001; ^3^Second Hospital of Shanxi Medical University, Shanxi, China 03001

## Abstract

Wiskott-Aldrich syndrome (WAS) is a rare X-linked recessive genetic disease characterized by clinical symptoms such as eczema, thrombocytopenia with small platelets, immune deficiency, prone to autoimmune diseases, and malignant tumors. This disease is caused by mutations of the *WAS* gene encoding WASprotein (WASP). The locus and type of mutations of the *WAS* gene and the expression quantity of WASP were strongly correlated with the clinical manifestations of patients. We found a novel mutation in the *WAS* gene (c.931 + 5G > C), which affected splicing to produce three abnormal mRNA, resulting in an abnormally truncated WASP. This mutation led to a reduction but not the elimination of the normal WASP population, resulting in causes X-linked thrombocytopenia (XLT) with mild clinical manifestations. Our findings revealed the pathogenic mechanism of this mutation.

## 1. Introduction

**Figure 1:**
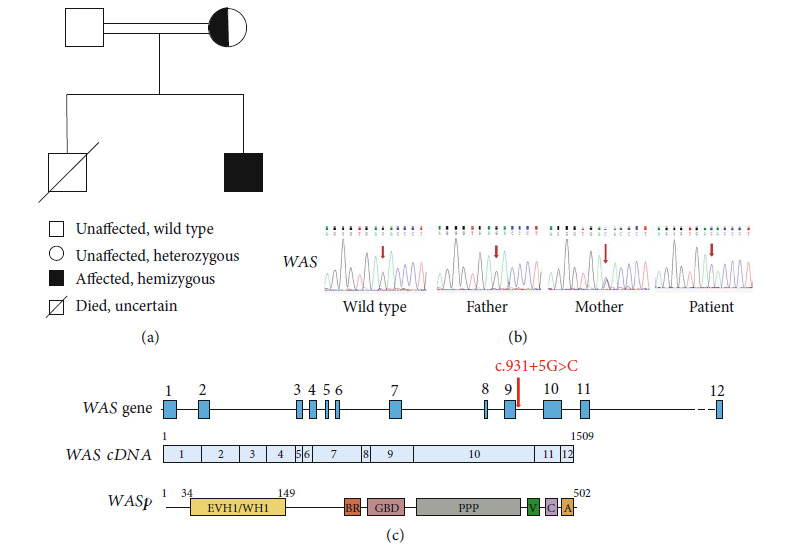
Family pedigree and mutation in *WAS*. (a) Family pedigree. (b) Sanger sequencing chromatograms depicting the c.931 + 5G > C (splicing) hemizygous mutation in *WAS* from the patient and the heterozygous mutation in his mother. (c) *WAS* gene showing the location of the patient's mutation indicated by the arrow. cDNA organization of *WAS* coding sequence and WASP structure. Boxes with numbers represent exons. Schematic diagram of WASP functional domains [4].

Wiskott-Aldrich syndrome (WAS) is a rare X-linked recessive genetic disease characterized by clinical symptoms such as eczema, thrombocytopenia with small platelets, immune deficiency, prone to autoimmune diseases, and malignant tumors [1]. This disease is caused by mutations of *WAS* gene encoding WASprotein (WASP), located on the X chromosome (XP11.22-11.23). This gene contains 12 exons, encoding WASP which is a 502-amino acid (a.a.) multidomain protein that is predominantly expressed in hematopoietic cells except for erythroid cells [2, 3]. It contains an N-terminal Ena-Vasp/WASP homology domain 1 (EVH1/WH1), followed by a basic region (BR), a GBD, a proline-rich region (PPP), and a G-actin binding verprolin homology (V) domain (Figure 1) [3, 4]. WASP is a multifunctional protein primarily involved in actin polymerization, signaling pathways, and cytoskeletal rearrangement, which plays a key role in monocytes and macrophage migrating and phagocytosis [5, 6]. Thus, complete or partial WASP deficiency results in tissue macrophage dysfunction, leading to recurrent infections and bleeding tendencies [7].

The locus and type of mutations of the *WAS* gene and the expression quantity of WASP were found to be significantly associated with the clinical manifestations of patients [8, 9]. Since the discovery of the *WAS* gene in 1994, more than four hundred mutations in *WAS* genes have been reported, the most common of which are missense and splice site mutations [4, 8]. Patients typically experience a reduction in the quantity or a truncated form of WASP [10]. These mutations lead to the expression of defective WASP, frequently causing the X-linked thrombocytopenia (XLT) phenotype, sometimes with only intermittent thrombocytopenia. XLT is a slightly milder form of the disease and other Wiskott-Aldrich syndrome complications, such as eczema and immune dysfunction, are typically mild or absent [11]. XLT is primarily characterized by thrombocytopenia, featuring small platelets, and may present mild infections and eczema [12, 13].

In this paper, we show that this novel splicing mutation (c.931 + 5G > C) produces three abnormal mRNA, resulting in a variety of abnormal truncated proteins, which result in a reduction of the WASP and thus cause X-linked thrombocytopenia (XLT), reveals its pathogenic mechanism from the perspective of molecular biology.

## 2. Material and Methods

### 2.1. Subjects

With the approval of the Ethics Committee of the Second Hospital of Shanxi Medical University ((2022) YX No. (046)), peripheral blood samples were collected from the patient, his parents, and healthy controls. The study was conducted in the Laboratory of Hematology, the Second Hospital of Shanxi Medical University. With the consent of the patient and his parents, genetic tests and analyses were performed.

### 2.2. Genetic Analysis

Genomic DNA (gDNA) was extracted from peripheral blood samples using the Gentra Puregene Blood Kit (Qiagen, Hilden, Germany). BioTek Cytation3 is a multifunctional cell imaging detection microplate reader for assessing the concentration and purity of extracted DNA. The target region of *WAS* DNA was amplified with Taq DNA polymerase (TaKaRa, Japan) using forward primer 5′ TCGCCTTATTCCTCTACTCC 3′ and reverse primer 5′ AGAACGACCCTTGTTACCC 3′. The amplified polymerase chain reaction (PCR) products were subjected to Sanger sequencing commercially. The mutation identified by next-generation sequencing (NGS) was confirmed by Sanger sequencing. The sequence was compared with the reference sequence NG_007877.1 of *WAS* published at the National Centre for Biotechnology Information (NCBI) by using SnapGene Viewer software. The MutationTaster was used to predictively analyze the pathogenicity of the mutation.

### 2.3. RNA Extraction and Reverse Transcription-PCR (RT-PCR)

RNA was extracted from peripheral blood using RNAiso Plus (TaKaRa, Japan). Prime Script RT Master Mix (TaKaRa, Japan) was used for reversed transcription to obtain cDNA. Using Taq DNA polymerase (TaKaRa, Japan) with forward primer 5′ GTGGGACCCCCAGAATGGATTTGAC 3′ and reverse primer 5′ CAGAACGACCCTTGTTACCC 3′ amplifies exons 8-10 of *WAS* cDNA. Actin beta cDNA, a housekeeping gene, was used as an internal control and amplified with forward primer 5′ TCATCACCATTGGCAATGAG 3′ and reverse primer 5′ CACTGTGTTGGCGTACAGGT 3′. The effect of this mutation on transcription is detected by RT-PCR. The PCR products were identified by electrophoresis to distinguish the bands of interest and finally were purified by the gel extraction kit (Omega Bio-Tek, China) for Sanger sequencing commercially. The sequence was compared with the reference sequence NM_000377.3 of *WAS* published at the NCBI by using SnapGene viewer software.

### 2.4. RNA Extraction and Real-Time PCR (RT-qPCR)

RNA was extracted from peripheral blood using RNAiso Plus (TaKaRa, Japan). PrimeScript™ RT reagent Kit with gDNA Eraser (TaKaRa, Japan) was used for reversed transcription to obtain cDNA. TB Green Premix Ex Taq II (TaKaRa, Japan) was used to amplify exons 8-10 of *WAS* cDNA with forward primer′ GTGGGACCCCCAGAATGGATTTGAC 3′ and reverse primer 5′ GGGCGGCGGAAGTGGCTCCT 3′. Intron 9-exon10 of *WAS* cDNA was amplified with forward primer 5′cactcagtccttatgggagcacc3 ′ and reverse primer 5′ GGGCGGCGGAAGTGGCTctg3′. Actin beta cDNA was used as a control and amplified with forward primer 5′ TCATCACCATTGGCAATGAG 3′ and reverse primer 5′ CACTGTGTTGGCGTACAGGT 3′. RT-qPCR is used to detect the relative level of abnormal mRNA produced by this mutation and its effect on normal mRNA levels. The relative quantity was quantified by Applied Biosystems 7500 Real-Time PCR System. The data are multiple biological replicates from (ideally) patient samples, with each experiment including three technical replicates. The one-way ANOVA was used to compare the differences between groups using the GraphPad Prism 9.0 software.

### 2.5. Flow Cytometry (FCM) Analysis of the Expression Levels of WASP

Flow cytometry was used to detect the expression of WASP in peripheral blood monocytes. Using human lymphocyte separation medium (Solarbio, Beijing, China) density gradient centrifugation, PBMCs were isolated from the peripheral blood of the patient and his parents. Using IntraPrep Permeabilization Reagent (Beckman Coulter, A07803), cells were fixed for 15 minutes and permeabilized for 15 minutes, respectively. Next, add the 1 : 100 dilution of rabbit anti-WASP (Abcam, ab75830, UK) to the samples and incubate for 30 min at room temperature. Finally, add 1 : 2000 diluted goat anti-rabbit IgG H&L (fluorescein isothiocyanate; Abcam, ab205718) and incubate for 25 min in the dark. After the cells were centrifuged and resuspended, we used the NAVIOS Flow Cytometer (Beckman Coulter) to detect the expression of WASP, and the data were analyzed by NAVIOS software.

### 2.6. Protein Structure Simulation

Using the AlphaFold Protein Structure Database (https://alphafold.com/), we obtained the PDB sequence of the WASP. Pymol software was used to predict the protein model of this mutation and analyze the effect of this mutation on the protein structure.

## 3. Results

### 3.1. Patients' Clinical Presentations and Immunologic Findings

The patient, a male, 26 years old, was admitted to the outpatient department of our hospital because of low platelet, easy-to-produce ecchymosis, and long-term mild eczema. After the patient was born, his parents discovered that he had bruises on his two lower limbs and chest, which worsened after being bumped. When the patient was 1 year old, he suffered epistaxis after a fall, then epistaxis often appeared. The patient is without fever, cough, melena, or hematemesis, there is no history of eczema. But the patient appeared fever, cough, or bruising after vaccination. Symptomatic treatment was administered, but no diagnosis was established. When the patient was 7 years old, his platelet count was 31 × 10^9^/L. The doctor prescribed three tablets of prednisone per day. After 3 months of continuous administration, the platelets rose to 100 × 10^9^/L. But then decreased to 20 − 40 × 10^9^/L after prednisone withdrawal. The patient was treated with ITP after multiple medical visits. The short-term effect of the treatment was favorable, although there was no significant improvement in the long-term effect. At the age of 12, the patient was suspected of Wiskott-Aldrich syndrome (WAS) at the Beijing Children's Hospital affiliated with Capital Medical University; however, a definitive diagnosis was not provided. The patient sought further diagnosis and genetic counseling by coming to our hospital. Genetic tests and analyses were conducted with the consent of the patient and his parents.

Laboratory studies showed severe thrombocytopenia and a reduced decreased mean platelet volume (MPV) in the patient. The quantities of red blood cells and levels of hemoglobin were within normal ranges. The white blood cell counts of the patient exhibited a slight elevation. The absolute number of NK cells went up. Thrombocytopenia with small platelets was the most notable among all the patient's outcomes. Notably, the lymphocyte subsets and functions in the patient showed no significant abnormalities (Table 1).

### 3.2. Genetic Analysis of the Patient

To determine the genetic defect in the family, we used NGS to identify the gene defect in this patient. Then, we confirmed the mutation by Sanger sequencing. One hemizygous mutation was detected on the X chromosome and carried a novel hemizygous mutation (c.931 + 5G > C) in the *WAS* gene. The Human Gene Mutation Database (HGMD) and the ClinVar both include no records of this mutation. This mutation was most likely the pathogenic mutation that contributed to the patient's clinical phenotype. The sequencing results of his parents indicated that his mother also carried a c.931 + 5G > C heterozygous mutation (Figure 1), while no mutations were detected in his father (Figure 1). This mutation that occurred in the patient was inherited from his mother, which was consistent with the inheritance mode of WAS (X-linked recessive inheritance). Based on the above results, we speculated that the patient suffered from WAS and his mother was an asymptomatic carrier of the *WAS*c.931 + 5G > C mutation. Unfortunately, his older brother died at a young age due to severe bleeding from a car accident (Figure 1), and it was presumed that he also suffered from WAS.

This mutation was predicted to be pathogenic by the online software MutationTaster [14]. The predicted results show that it is harmful, which may affect splicing and cause disease and the mutation site had high base conservation. This mutation was not reported in patients with WAS/XLT, and the clinical picture of the patient is consistent with XLT, so we concluded that this mutation is responsible for his thrombocytopenia.

### 3.3. Effect of the Novel Splicing Mutation on RNA Level

**Figure 2:**
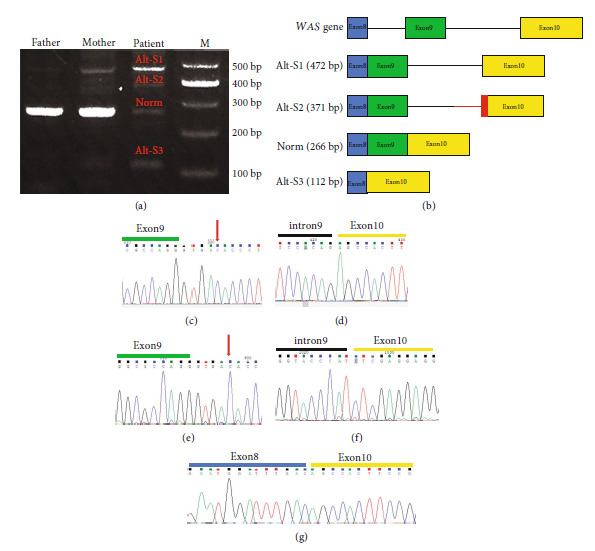
The splicing situation of mRNA in the *WAS* gene in the patient and his parents. (a) RT-PCR electrophoresis identification map. Lane 1: RT-PCR products from father. Lane 2: RT-PCR products from mother. Lane 3: RT-PCR products from the patient. M: DNA maker. (b) Schematic diagram of *WAS* gene and the patient's four transcripts. Three abnormal spliced mRNAs of the patient. The red area is the missing sequences. (c, d) Alt-S1 Sanger sequencing. (e, f) Alt-S2 Sanger sequencing. (g) Alt-S3 Sanger sequencing. The location of the patient's mutation is indicated by the arrow.

The novel splicing mutation may impact the exon 9 splice acceptor location, causing exons 8 to 10 to be spliced incorrectly. To test this theory, we took RNA from peripheral blood taken from the patient, his parents, and one control, reverse-transcribed, and then used RT-PCR primers that were specific for exons 8 to 10 of *WAS* to amplify the cDNA. This revealed the presence of three *WAS* aberrant splicing bands in the patient different from his parents and health controls. The results showed that the patient produced three abnormal mRNA, and his mother who carried this mutation also produced a small number of abnormal mRNA (Figure 2). The splicing of three abnormal mRNA sequences was tested using Sanger sequencing and analyzed with SnapGene Viewer software. The Alt-S1 sequence showed that intron 9 was retained. Although the Alt-S2 sequence also retained intron 9, the 3′ end of intron 9 lost 74 nt, and the 5′ end of exon 10 lost 27 nt. The Alt-S3 sequence is missing exon9 (Figure 2). These results demonstrate that the c.931 + 5G > C mutation affects the splicing of *WAS* mRNA resulting in exon hopping and partial intron retention to probably produce truncated protein and reduce WASP expression.

### 3.4. RNA Extraction and RT-qPCR

**Figure 3:**
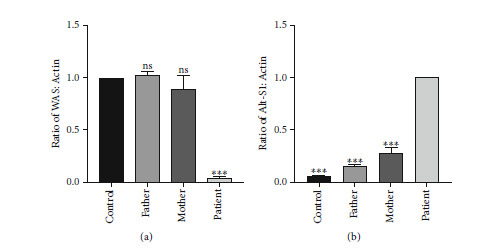
mRNA relative quantity. (a) Norm *WAS* mRNA relative quantity. (b) Alt-S1 mRNA relative quantity. There was a significant difference between the patient and the normal control, and the difference was statistically significant (*P* < 0.001). The one-way ANOVA: ns, not significant, ^∗^*P* < 0.05, ^∗∗^*P* < 0.01, ^∗∗∗^*P* < 0.001.

By RT-PCR electrophoresis identification map, we identified three abnormal mRNA in the patient and the band of Alt-S1 is the brightest. We used RT-qPCR to amplify intron 9-exon 10 and exons 9-10 of *WAS* cDNA to detect the relative level of Alt-S1 and *WAS* norm mRNA to explain the difference in *WAS* mRNA expression in the patient. Results were statistically analyzed using the GraphPad Prism V8.0 software. His parents' norm *WAS* mRNA relative quantity showed no significant difference compared with the normal control. However, the norm *WAS* mRNA relative quantity of the patient significantly decreased (*P* < 0.001) (Figure 3(a)). The Alt-S1 mRNA relative quantity of the patient taken as the control, and the normal control and his parents were far less than the patient (*P* < 0.001) (Figure 3(b)). There was a significant difference between the patient and the normal control, and the difference was statistically significant (*P* < 0.001).

### 3.5. Flow Cytometry Analysis of Expression Levels of WASP

**Figure 4:**
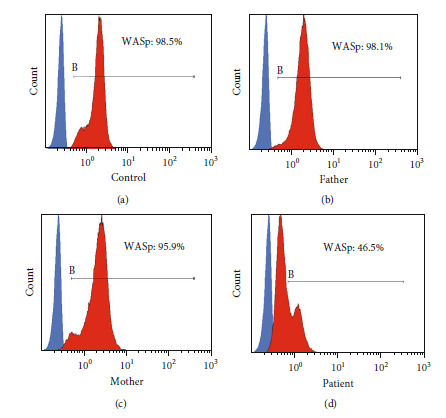
Flow cytometry analysis expression levels of WASP. (a) Control. (b) Father. (c) Mother. (d) Patient.

We conducted a quantitative assessment of WASP expression levels in PBMCs from all participants using flow cytometry to further confirm the pathogenicity of the novel mutation. WASP expression in the patient was only slightly downregulated (45.9%), while his parents' WASP expression was 95.9% and 98.1%, respectively (Figure 4). The patient's PBMC had a low level of WASP expression, as shown in the above results. The results showed that the mutation reduced but did not eliminate the expression of WASP (Figure 4), resulting in the patient with a milder phenotypic XLT.

### 3.6. Protein Structure Simulation

**Figure 5:**
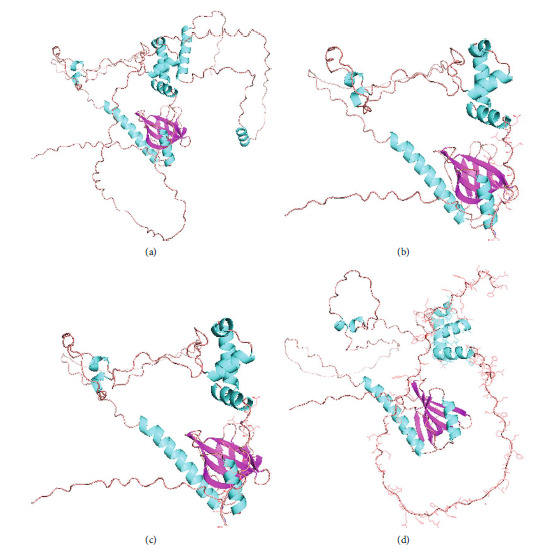
Pymol software analysis WASP of the patient. (a) Norm WASP. (b) Alt-S1. (c) Alt-S2. (d) Alt-S3.

Early termination of amino acid sequence may occur due to abnormal mRNA splicing. Using the AlphaFold Protein Structure Database (https://alphafold.com/), we obtained the PDB sequence of the WASP (AF-P42768-F1). Pymol software was used to predict the protein model encoded by the three abnormal transcripts. We obtained three WASP with abnormal C-terminal allosteric truncation obtained by the constructed protein model (Figure 5). We believe that these abnormally truncated proteins not only function abnormally but also degrade prematurely, so this mutation causes a decrease in the normal WASP population and therefore causes this clinical symptom in the patient.

### 3.7. Treatments and Outcomes

The patient only had thrombocytopenia, small platelets, and long-term mild eczema. The patient was identified as having WAS and assigned the classification XLT (patient, a score of 1) based on the collected clinical data, genetic results, and the expression level of WASP, the diagnostic criteria for primary immunodeficiencies published in 1999, as well as a 5-point clinical severity scoring system of WAS [15].

Hematopoietic stem cell transplantation (HSCT) is the most effective method to cure WAS at present; gene therapy is still in the experimental stage, and its safety needs to be further improved [16, 17]. HSCT in XLT has to be decided on an individual patient basis [18]. HSCT requires a full preoperative evaluation of the patient, and the benefits of a potential cure must be carefully weighed against the immediate hazards and long-term effects of this treatment [19]. The patient has no other symptoms except stubborn thrombocytopenia with reduced platelet volume and mild eczema. Conservative treatment is under consideration, and regular physical examination is recommended [20].

## 4. Discussion

In this study, we report a novel pathogenic *WAS* gene mutation (c.931 + 5G > C (splicing)) causing XLT with mild clinical manifestations in a family from China and revealed the pathogenic mechanism of this mutation. We demonstrate that the c.931 + 5G > C mutation affects normal splicing of *WAS* mRNA, resulting in exon hopping and partial intron retention to produce a truncated protein and reduce WASP expression. We provide mechanistic proof of the variant's biological effect. But the functional consequence of this mutation is uncertain. The C-terminal VCA domain folds back and binds to the GTPase binding domain (GBD), maintaining the protein in an inactive state. Loss of the C-terminus may prevent the autoinhibition caused by the VCA-GBD interaction and lead to an ACTIVE conformation, although lacking the acidic sequence which allows interactions with Arp2/3. Given the patient's phenotype, it is more likely that there is leaky splicing occurring which is allowing some WT splice product, as suggested by the RT-PCR, and mostly mutant product which may undergo nonsense-mediated decay, suggested by the decreased expression of WAS transcript. Flow cytometry clearly shows a decreased level of WASP. The functional consequence of this mutation needs to be further investigated. The mutation is closely related to the pathogenesis of WAS milder phenotype-XLT, which coincides with the clinical phenotype of the patient. Clinical phenotypes caused by deletion or low expression of WASP due to pathogenic mutations in the *WAS* gene include typical WAS, X-linked thrombocytopenia (XLT), and X-linked neutropenia (XLN) [8]. Our report broadens the pathogenic mutation spectrum of the *WAS* gene and stresses the value of molecular genetic testing in the diagnosis of WAS due to the vast spectrum of the disease phenotype of WAS. The time it takes to diagnose more patients from the same family or the same place is shortened by using genetic testing, which is particularly cost-effective when screening members of the same family.

Patients with Wiskott-Aldrich syndrome who exhibit a milder phenotype are clinically classified as XLT. This classification is primarily characterized by thrombocytopenia with small platelets and may also present mild infections and eczema. Adult patients with XLT are often misclassified as chronic immune thrombocytopenic purpura (ITP), so the differential diagnosis of ITP is crucial [21]. ITP can occur in infancy and can impact individuals of both genders. There is no family history of hemorrhagic disease, normal platelet volume, detectable antiplatelet antibodies, no persistent eczema or infectious susceptibility, and good response to hormone and high-dose intravenous immunoglobulin therapy. In certain children diagnosed with WAS or XLT, the initial use of hormone therapy or intravenous immunoglobulin (IVIG) has some effect, and the subsequent effect should be paid attention to. The significance of bone marrow puncture in differentiating the two is limited. Therefore, male, early onset, stubborn thrombocytopenia accompanied by reduced platelet volume are important indications for protein expression analysis and gene analysis of WAS.

In conclusion, Wiskott-Aldrich syndrome is a rare X-linked disorder that predominantly affects males. Male patients with congenital thrombocytopenia and small platelets must have their *WAS* gene mutation and WASP expression evaluated as soon as possible using molecular genetic techniques and protein detection [22]. In this study, we identified a novel pathogenic *WAS* gene mutation (c.931 + 5G > C (splicing)) causing XLT with mild clinical manifestations. The pathogenicity of this locus has not been reported in the Human Gene Mutation Database and the ClinVar database, and the pathological significance of this locus has not been reported in the literature. We report this mutation for the first time, and it is also the first time to verify that this mutation decreased the expression of WASP and provided more evidence supporting the pathogenicity of the mutation [23]. Researching and reporting novel pathogenic mutations in the *WAS* gene will broaden the spectrum of mutations associated with this gene, facilitating genetic counseling and prenatal diagnosis [24]. This study has expanded the gene mutation database of WAS syndrome and has offered new insights for more accurate identification of mutation types and clinical treatment of XLT patients.

## Figures and Tables

**Table 1:** Laboratory investigations of the WAS patient.

Test items	Result (normal)
Platelets (cells/L × 10^−9^)	13 ↓ (125~350)
Mean platelet volume (fL)	6.60 ↓ (9.20~12.00)
Width of platelet volume distribution (fL)	16.00 ↑ (9.60~12.00)
Thrombocytocrit (%)	0.01 ↓ (0.19~0.39)
Red blood cells (cells/L × 10^−12^)	5.18 (4.30~5.80)
Hemoglobin (g/L)	156.0(130.0 ~ 175.0)
White blood cell numbers (cells/L × 10^−9^)	11.10 ↑ (3.50~9.50)
Neutrophil numbers (cells/L × 10^−9^)	6.88 ↑ (1.80~6.30)
Neutrophil percentage (%)	61.73 (40.00~75.00)
Mononuclear cell numbers (cells/L × 10^−9^)	0.86 ↑ (0.10~0.60)
Mononuclear cell percentage (%)	7.68 (3.00~10.00)
Lymphocyte numbers (cells/L × 10^−9^)	3.25 ↑ (1.10~3.20)
Lymphocyte percentage (%)	29.14 (20.00~50.00)
CD3+ (%)	65.50 (60.00~80.00)
Th (CD3 + CD4+) (%)	36.90 (30.09~40.41)
Ts (CD3 + CD8+) (%)	23.80 (20.74~29.42)
Th/Ts (%)	1.55 (0.98~1.94)
CD3 − CD16 + /CD56+ (%)	30.50 ↑ (8.10~25.60)
CD3 + CD16 + /CD56+ (%)	0.40 (≤5.00)
CD19+ (%)	7.90 ↓ (9.00~15.00)
IgA (g/L)	4.07 (0.82~4.53)
IgG (g/L)	7.97 (7.51~15.60)
IgM (g/L)	0.20 ↓ (0.46~3.04)
Control (%)	0.40 (0-3.00)
PlaIgA (%)	1.50 (0-10.0)
PlaIgG (%)	2.60 (0-10.0)
PlaIgM (%)	1.30 (0-10.0)

## Data Availability

The data used to support the findings of this study are available from the corresponding authors upon request.
